# Blend Sign Is a Strong Predictor of the Extent of Early Hematoma Expansion in Spontaneous Intracerebral Hemorrhage

**DOI:** 10.3389/fneur.2020.00334

**Published:** 2020-05-19

**Authors:** Mingyue Zhang, Jie Chen, Chenyi Zhan, Jinjin Liu, Qian Chen, Tianyi Xia, Tingting Zhang, Dongqin Zhu, Chao Chen, Yunjun Yang

**Affiliations:** Department of Radiology, The First Affiliated Hospital of Wenzhou Medical University, Wenzhou, China

**Keywords:** intracerebral hemorrhage, extent of hematoma expansion, functional outcome, blend sign, computed tomography

## Abstract

**Background and Purpose:** It is unclear which imaging marker is optimal for predicting the extent of hematoma expansion (EHE). We aimed to compare the usefulness of the blend sign (BS) with that of other non-contrast computed tomography (NCCT) markers for predicting the EHE in patients with spontaneous intracerebral hemorrhage (sICH).

**Methods:** Patients with sICH admitted to our Neurology Emergency Department between September 2013 and January 2019 were enrolled. The EHE was calculated as the absolute increase in hematoma volume between baseline and follow-up CT (within 72 h). The EHE was categorized into four groups: “no growth,” “minimal change” (≤5.1 ml), “moderate change” (5.1–12.5 ml), and “massive change” (>12.5 ml). Univariate and multivariate analyses were performed to investigate the relationship between the NCCT markers [BS, black hole sign (BHS), satellite sign, and island sign] and the EHE.

**Results:** A total of 1,111 sICH patients were included (median age: 60 years; 66.5% males). Multiple linear regression analysis showed that the presence of the BS and BHS was independently associated with the EHE, after adjusting for confounders (*P* < 0.001 and *P* = 0.003, respectively). The presence of the BS and BHS was positively correlated with growth category (*r* = 0.285 and *r* = 0.199, both *P*s < 0.001). The BS demonstrated a better predictive performance for the EHE than did the BHS [area under the curve (AUC): 0.67 vs. 0.57; both *P*s < 0.001].

**Conclusions:** In patients with acute sICH, the BS showed a better performance in predicting the EHE compared with other NCCT markers. This imaging marker may help identify patients at a high risk of significant hematoma expansion and may facilitate its early management.

## Introduction

Spontaneous intracerebral hemorrhage (sICH) has become the leading cause of disability and death in China, and early hematoma expansion (HE) is an independent risk factor for early neurological deterioration and long-term functional outcomes (1). In a large meta-analysis, intracerebral hemorrhage (ICH) baseline volume, non-contrast computed tomography (NCCT) timing, and antiplatelet and anticoagulant use were found to be independent predictors of intracerebral hematoma growth (2). HE occurs in 20–40% of all patients with sICH (3, 4), and previous reports have identified multiple factors that are associated with clinical outcomes following sICH, including age, baseline hematoma volume, HE, the presence of a neurological deficit, intraventricular hemorrhage (IVH), and hematoma location (4–6). Among these factors, hemorrhage volume is a significant predictor of poor outcome in patients with sICH (7), and this is an area of ongoing research. Davis et al. found that an increase in the hematoma volume by 10% increases the risk of death at 90 days by 5% and poor outcome by 16–18% (4). Conversely, an approximately 2–4 ml reduction in hematoma volume was associated with a 20–30% reduction in the risk of death (8). Thus, increased hemorrhage volume has been a major focus for potential therapeutic targets, as it is the only modifiable factor which is present in the majority of patients (9–12). Therefore, it is important to find markers associated with the extent of hematoma expansion (EHE).

The computed tomography angiography (CTA) spot sign is regarded as a useful predictor for HE and poor functional outcomes (13–17). However, CTA is not routinely used in many institutions (18). Recently, several NCCT markers have shown the ability to predict HE (19), such as the blend sign (BS) (20), black hole sign (BHS) (21), satellite sign (22), and island sign (23). Which imaging marker is better at predicting the EHE remains unclear. Therefore, the purpose of this study was to compare their performances in predicting the EHE.

## Methods

### Study Population

We retrospectively evaluated consecutive sICH patients who were admitted to our Neurology Emergency Department between September 2013 and January 2019. The eligibility criteria for our study included patients aged >18 years old whose baseline NCCT and follow-up NCCT were performed within 6 and 72 h from symptom onset, respectively. We used the following exclusion criteria: (1) hemorrhage secondary to tumor, aneurysm, trauma, arteriovenous malformation, or hemorrhagic infarction; (2) surgical intervention for the hematoma performed prior to the follow-up CT scan; (3) multiple cerebral hemorrhages or primary IVH; and (4) a lack of hospitalization data and follow-up CT. In addition, macrovascular causes were detected using CTA. CTA was not performed routinely in all patients, but at the discretion of the clinical team. The management of patients followed the recommendations provided by the American Heart Association/American Stroke Association (AHA/ASA) guidelines. Our study was approved by the ethics committee of the First Affiliated Hospital of Wenzhou Medical University.

### Clinical Data

Clinical data such as age, sex, medical history (including smoking, alcohol consumption, and diabetes mellitus), admission platelet count, admission systolic blood pressure, time from onset to baseline CT (h), time from baseline CT to follow-up CT (h), and Glasgow Coma Scale (GCS) score were retrospectively collected. The Glasgow Outcome Scale (GOS) score was evaluated at discharge, and patients were dichotomized into favorable outcome (4–5 points) and poor outcome (1–3 points).

### Image Acquisition and Analysis

The baseline and follow-up CT scans (120 kVp, 80 mA; section thickness, 5 mm) were performed using a standard clinical protocol. All CT images were acquired from the picture archiving and communication system (PACS) and saved in DICOM format for further evaluation. Volumetric calculation of the hematoma was completed through three-dimensional reconstruction of the regions of interest obtained by manually depicting the lesion circumference in multiple successive layers on baseline and follow-up NCCT imaging. All measurements were performed in a blinded fashion. Two experienced radiologists, who were blinded to the patients' clinical data, independently evaluated all baseline and follow-up CT images. Any disagreement between the two radiologists was resolved by consensus. Hematoma location and volume, the presence of the intraventricular extension (16), BHS (21), BS (20), island sign (23), and satellite sign (22) was assessed. Hematoma location was divided into deep, lobar, brain stem, and cerebellum (24), and deep ICH was defined as hematoma involving the thalamus or basal ganglia. Baseline hematoma volume was divided into four categories: <10, 10–20, 20–30, and ≥30 ml. The EHE was defined as an increase in hematoma volume between baseline and follow-up CT. The extent of hematoma growth was classified into four “clinically meaningful” grades: “no growth,” “minimal change” (≤5 ml), “moderate change” (5.1–12.5 ml), and “massive change” (>12.5 ml) (8). In addition, follow-up CT was performed routinely within 72 h, but the exact timing of the follow-up CT was at the discretion of the clinical physician and time to deterioration of patients.

### Statistical Analyses

Statistical analyses were performed using R software (version 3.6.0; The R Foundation for Statistical Computing, Vienna, Austria) and SPSS (version 24.0; IBM Corp., Armonk, NY, USA). Continuous variables were described as medians or means; categorical variables were expressed in percentages. We performed exploratory simple linear regression analysis to explore the association between patient characteristics and absolute growth, followed by multiple linear regression to determine the independent predictors of absolute hematoma growth. Variables with a *P* < 0.05 in univariate regression analyses were included in the multiple linear regression. Previous use of antiplatelet and anticoagulant medications, which were thought to be independent predictors associated with HE, was forced into the model. The absolute growth and imaging markers were compared using chi-square or Fisher's exact test and Student's *t*-test or Mann–Whitney *U*-test, as appropriate. The correlation analysis was performed using Kendall's tau-*b* test. Receiver operating characteristic analysis was performed to evaluate the value of the BS and BHS in predicting HE (>12.5 ml). Multivariate logistic regression analysis was performed to investigate whether the EHE was an independent predictor of poor outcome in patients with ICH, adjusting for predictors of poor outcome that were previously used in the INTERACT1 study (8). The amount of missing data was low (<5%), and patients with missing values were not involved in the multivariate logistic regression analysis. *P* < 0.05 was considered statistically significant.

## Results

A total of 1,111 sICH patients (739 males and 372 females) were included in our study. The median age of the patients was 60.90 years [interquartile range (IQR) 51–70 years]. Baseline characteristics are shown in (Table 1). Exploratory simple linear regression analysis revealed that sex, baseline volume, location of hemorrhage, time from onset to baseline CT, time from baseline CT to follow-up CT, systolic blood pressure, baseline GCS, and the presence of the satellite sign, BS, BHS, and island sign were all associated with the EHE (all *P*s < 0.05; Table 2). Multivariate analysis with multiple linear regression showed that the BS (*P* < 0.001) and BHS (*P* = 0.003) were independently associated with absolute growth after adjusting for sex, baseline volume, location of hemorrhage, time from onset to baseline CT, time from baseline CT to follow-up CT, systolic blood pressure, antiplatelet, anticoagulant, and baseline GCS (Table 3).

**Table 1:** Baseline characteristics of the study cohort.

**Demographic characteristics**	**Total (*n* = 1,111)**
Age, years, median (IQR)	60.9 (51–70)
Male sex, *n* (%)	739 (66.5)
Systolic blood pressure, median (IQR)	161 (144–177)
**Clinical characteristics**	
History of diabetes mellitus, *n* (%)	140 (12.7)
History of smoking, *n* (%)	350 (31.8)
History of alcohol intake, *n* (%)	348 (31.6)
Antiplatelet, *n* (%)	19 (1.7)
Anticoagulant, *n* (%)	8 (0.7)
Platelet count, ×10^3^/μl, median (IQR)	202.5 (165–242)
**Location**	
Lobar, *n* (%)	82 (7.4)
Deep, *n* (%)	951 (85.6)
Brain stem, *n* (%)	49 (4.4)
Cerebellum, *n* (%)	29 (2.6)
GCS score, median (IQR)	13 (10–15)
Time from onset to first CT (h), median (IQR)	3 (2–4)
Time from the first to the follow-up CT (h), median (IQR)	19.5 (12–29.5)
Discharge GOS <4, *n* (%)	748 (67.3)
Baseline volume, median (IQR)	16.4 (8.9–27.1)
Presence of IVH, *n* (%)	386 (34.7)
Black hole sign, *n* (%)	173 (15.6)
Blend sign, *n* (%)	169 (15.2)
Satellite sign, *n* (%)	420 (37.8)
Island sign, *n* (%)	230 (20.7)

*Continuous variables are presented as median (IQR); categorical variables are expressed in n (%)*.

*CT, computed tomography; GCS, Glasgow Coma Scale; GOS, Glasgow Outcome Scale; IQR, interquartile range; IVH, intraventricular hemorrhage*.

**Table 2:** Simple linear regression analysis of predictors of the extent of early hematoma expansion.

**Predictors**	***B***	**β**	**95% CI of *B***	***P*-value**
Sex, male	1.923	0.084	0.575–3.271	0.005
Age	−0.011	−0.012	−0.062–0.041	0.685
Systolic blood pressure	−0.036	−0.087	−0.061 to −0.011	0.004
**Medical history**				
History of diabetes mellitus	0.210	0.006	−1.715–2.134	0.831
History of smoking	0.805	0.035	−1.571–2.181	0.215
History of alcohol intake	0.805	0.032	−0.766–2.375	0.315
Antiplatelet	−2.628	−0.031	−7.552–2.296	0.295
Anticoagulant	23.039	0.180	15.608–30.470	<0.001
Platelet count	−0.010	−0.067	−0.019–0.001	0.026
Time from onset to first CT	−0.679	−0.090	−1.123 to −0.235	0.003
Time from the first to the follow-up CT	−0.127	−0.183	−0.167 to −0.087	<0.001
Location of hemorrhage	−0.993	−0.06	−0.968–0.018	0.046
Baseline hematoma volume	0.148	0.201	0.105 to 0.019	<0.001
Presence of IVH	0.515	0.023	−0.826–1.855	0.451
Baseline GCS score	−0.671	−0.203	−0.863 to −0.479	<0.001
Black hole sign	4.517	0.151	2.777–6.258	<0.001
Blend sign	6.969	0.231	5.240–8.699	<0.001
Satellite sign	1.924	0.086	0.612–3.236	0.004
Island sign	2.696	0.101	1.126–4.267	0.001

*B, unstandardized coefficients; β, standardized coefficients; 95% CI, 95% confidence interval for B; CT, computed tomography; GCS, Glasgow Coma Scale; IVH, intraventricular hemorrhage*.

**Table 3:** Multiple linear regression analysis of predictors of the extent of early hematoma expansion.

**Predictors**	***B***	**β**	**95% CI**	***P*-value**
Sex, male	1.729	0.078	0.465–2.993	0.007
Systolic blood pressure	−0.023	−0.056	−0.046–0.001	0.047
Antiplatelet	−0.406	−0.005	−4.784–3.971	0.855
Anticoagulant	22.651	0.188	15.924–29.378	<0.001
Time from onset to first CT	−0.608	−0.083	−1.018 to −0.199	0.004
Time from the first to the follow-up CT	−0.067	−0.101	−0.105 to −0.023	0.001
Location of hemorrhage	−0.439	−0.035	−1.132–0.255	0.855
Baseline hematoma volume	−0.001	−0.001	−0.052–0.050	0.976
Baseline GCS score	−0.528	−0.162	−0.722 to −0.333	<0.001
Black hole sign	2.524	0.087	0.861–4.188	0.003
Blend sign	5.658	0.195	3.958–7.358	<0.001
Satellite sign	0.247	0.011	−1.325–1.819	0.758
Island sign	1.915	0.074	−0.022–3.852	0.053

*Adjustment by sex, baseline volume, location of hemorrhage, time from onset to baseline CT, time from baseline CT to follow-up CT, systolic blood pressure, antiplatelet, anticoagulant, baseline GCS score*.

*B, unstandardized coefficients; β, standardized coefficients; 95% CI, 95% confidence interval for B; CT, computed tomography; GCS, Glasgow Coma Scale; IVH, intraventricular hemorrhage*.

**Figure 1:**
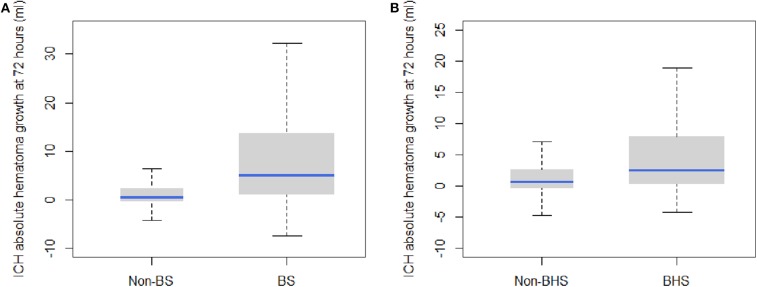
Box plots of hematoma growth between the baseline and 72-h CT scans depict a greater hemorrhage volume growth in **(A)** the BS vs. non-BS scans (*P* < 0.001) and **(B)** the BHS vs. non-BHS scans (*P* < 0.001). BHS, black hole sign; BS, blend sign; CT, computed tomography.

**Figure 2:**
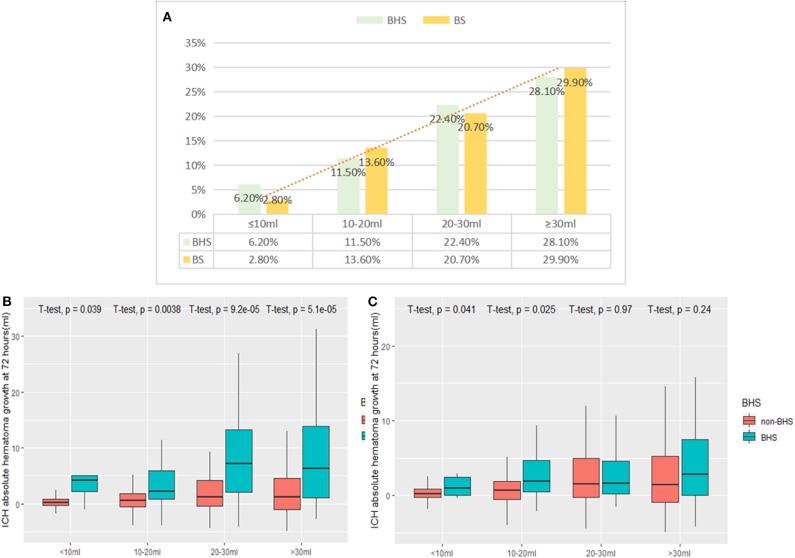
**(A)** shows the frequency of the BS and BHS, stratified by baseline hematoma volume (both *P*s < 0.001). Differences between **(B)** the BS and non-BS and **(C)** the BHS and non-BHS scans are shown in the figure. BHS, black hole sign; BS, blend sign.

The BS and BHS showed a statistically significant association with absolute growth on follow-up CT (Figure 1). Compared with the non-BS group, patients with the BS had a significantly higher median hematoma growth (0.45 [0.39–2.29] vs. 5.10 [0.98–13.91] ml, *P* < 0.001; Figure 1A). Median growth was higher in the BHS group than in the non-BHS group (2.46 [0.27–7.96] vs. 0.58 [−0.38 to 2.62] ml, *P* < 0.001; Figure 1B). Patients with small baseline hematoma volumes were less likely to present the BS or BHS (both *P*s < 0.001; Figure 2A). (Figure 2) demonstrates the presence of a significant difference in the amount of hematoma growth between patients in the BS and non-BS groups for all baseline hematoma volumes (all *P*s < 0.01; Figure 2B). When we compare the BHS and non-BHS groups, significant differences were only observed in those with a baseline hematoma volume of up to 20 ml (Figure 2C).

**Figure 3:**
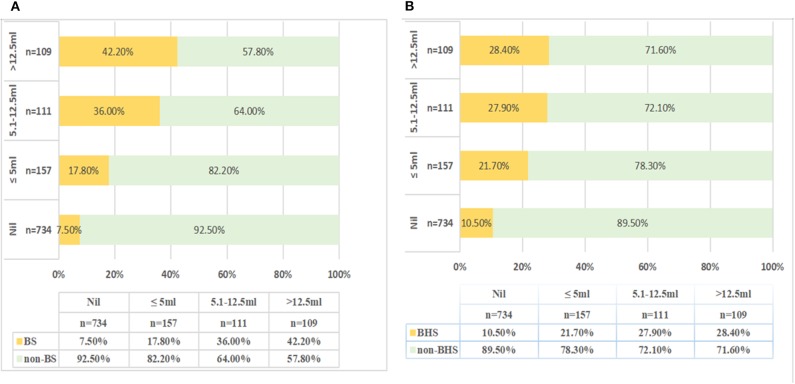
The proportions (%) of the BS **(A)** and BHS **(B)** in the four groups stratifying the extent of hematoma growth. The *x*-axis refers to the percentage of the BS or BHS (%). *P* < 0.001. BHS, black hole sign; BS, blend sign; Nil, no growth.

**Figure 4:**
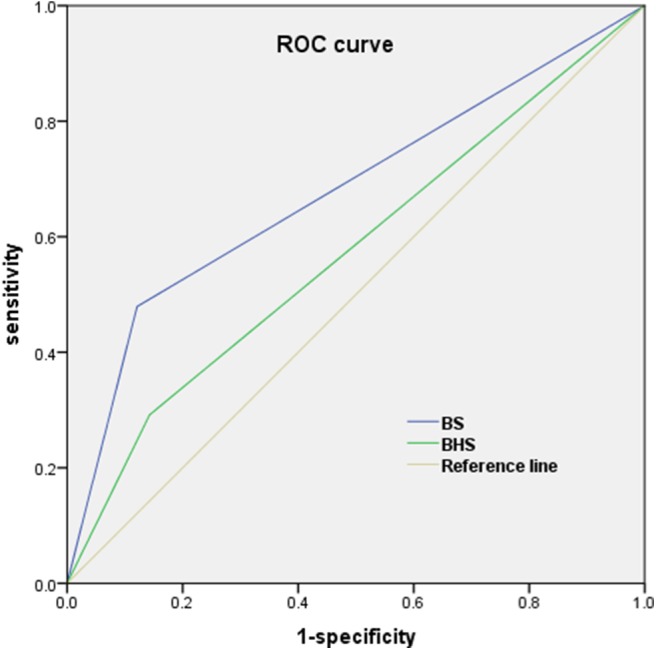
The receiver operating characteristic (ROC) curve of the BS and BHS were used for predicting HE (>12.5 ml). The area under the curve for the BS was 0.67 and that for the BHS was 0.57. BHS, black hole sign; BS, blend sign.

On moving to the categories of higher hematoma growth from nil to >12.5 ml, the frequency of the BS and BHS increased from 7.5 to 42.2% and from 10.5 to 28.4%, respectively (*P* < 0.001; Figures 3A,B). Compared with that in “no growth” (7.5%), the incidence of BS was significantly higher in the “minimal change” (17.8%) or “moderate change” (36%) groups (*P* < 0.001). The “massive change” group showed the highest incidence of the BS (42.2%). The receiver operating characteristic analysis showed that the area under the curve (AUC) of the BS was 0.67 (95% CI 0.62–0.74) and that of the BHS was 0.57 (95% CI 0.51–0.64) in predicting HE (Figure 4). In addition, the BS and BHS showed positive correlations with the baseline hematoma category (BS: *r* = 0.249, *P* < 0.001; BHS: *r* = 0.211, *P* < 0.001). The BS and BHS also had positive correlations with the hematoma growth category (BS: *r* = 0.285, *P* < 0.001; BHS: *r* = 0.199, *P* < 0.001). After known predictors of outcome were adjusted for, compared to that in patients with “no growth,” the change in hematoma growth was independently associated with higher odds of poor outcome following sICH (OR: 1.030 [95% CI 0.679–1.565], *P* = 0.888 for “minimal change,” and OR: 2.227 [95% CI 1.222–4.060], *P* = 0.009 for “moderate change”). Patients with “massive change” had the highest risk of a poor outcome (OR: 2.534 [95% CI 1.296–4.955], *P* = 0.007, Table 4).

**Table 4:** Multivariate logistic regression analysis of predicting poor outcome.

**Variable**	**Adjusted OR**	**95% CI**	***P*-value**
Sex, male	0.898	0.663–1.215	0.486
Age, year	1.016	1.004–1.029	0.009
Systolic blood pressure, mmHg	1.003	0.997–1.009	0.296
Time from onset to first CT, h	0.963	0.870–1.065	0.461
Time from the first to the follow-up CT, h	0.995	0.986–1.004	0.252
**Location of hemorrhage**			
Deep	1 [Reference]	1 [Reference]	1 [Reference]
Lobar	0.221	0.125–0.388	<0.001
Brain stem	0.108	0.048–0.240	<0.001
Cerebellum	0.340	0.143–0.809	0.015
Presence of IVH	1.191	0.867–1.635	0.281
Baseline hematoma volume, ml	1.044	1.029–1.059	<0.001
Baseline GCS score (≤ 8)	2.771	1.616–4.752	<0.001
**Extent of hematoma expansion**			
No growth (nil)	1 [Reference]	1 [Reference]	1 [Reference]
Minimal change (≤ 5 ml)	1.030	0.679–1.565	0.888
Moderate change (5.1–12.5 ml)	2.227	1.222–4.060	0.009
Massive change (>12.5 ml)	2.534	1.296–4.955	0.007

*OR, odds ratios; 95% CI, 95% confidence interval; CT, computed tomography; GCS, Glasgow Coma Scale; IVH, intraventricular hemorrhage*.

## Discussion

In our retrospective study of 1,111 patients with sICH, we compared the BS with several CT markers to predict the EHE. The main finding of this analysis was that the BS was better able to stratify the EHE than were other CT markers.

The INTERACT1 study showed an approximate linear relationship between HE and prognosis; every 1-ml increase in hematoma growth was associated with a 5% increase in the odds of a poor outcome (8). Studies have also shown that absolute HE definitions predict poor outcomes better than relative HE definitions (25). It is universally acknowledged that hematoma volume is the main determinant of poor outcome in patients with sICH (5, 6). A growth in hematoma volume of over 5 ml is clearly visible in clinical practice (8). In addition, baseline hematoma volume is a significant predictor of HE, which is directly related to both the final hematoma volume and the clinical outcome (5, 26). In our study, the incidence of the BS and BHS was both positively correlated with baseline hematoma volume category. With an increase in the baseline hematoma growth category, the frequency of the BS and BHS increased from 2.8 to 29.9% and from 6.2 to 28.1%, respectively. The HE volume was divided into four groups, and we identified a positive correlation between the incidence of the BS and the HE volume category. Conversely, in the BHS-positive patients, there was a plateau in the frequency of BHS above the 5.1-ml hematoma growth volume category. We found that the presence of the BS may indicate a greater risk in the occurrence of the final hematoma volume. The BS represented ongoing, active bleeding in the hematoma (20, 27), and its low incidence in patients with a small baseline hematoma indicates a stable hematoma and lower risk of expansion. In contrast, a large baseline hematoma volume probably reflects multifocal bleeding sources within the hematoma.

In our study, we found that when the baseline hematoma volume was divided into four categories (<10, 10–20, 20–30, and >30 ml), a linear correlation was detected between the baseline hematoma volume and the BHS and BS. Furthermore, we demonstrated that larger initial hematoma volumes are more frequent for those with BHS and BS, and they are more likely to develop toward a larger hematoma volume. Median growth of the hematoma was significantly greater in patients with BHS and BS than in CT marker-negative patients; this supports the current hypotheses regarding the pathophysiology of sICH expansion (28–31). These CT markers were consistent features for larger hematomas and reflect the natural history of HE in sICH.

The clinical value of the BHS and BS in predicting HE and secondary neurological deterioration has been confirmed in multiple studies (32–37). The principle underlying the BHS and BS is that the hematoma is heterogeneous. However, it is necessary to improve the reliability of the heterogeneous hematoma in predicting HE. Barras et al. (28) found that heterogeneous density of a hematoma independently predicted HE, with growth occurring on a continuous scale. In a retrospective analysis (38), the BS and spot sign were highly predictive of poor functional outcomes. Furthermore, the multivariate analysis confirmed the BS as a reliable predictor of poor functional outcome in patients with sICH, after adjusting for several known risk factors. Notably, Yu et al. (34) obtained similar results in predicting HE. Similar to the BS, there was a close relationship between the CTA spot sign and the BHS. However, Sporns (36) demonstrated a high degree of association between the BS and spot sign (κ = 0.701) and a moderate degree of association between the BHS and spot sign (κ = 0.424). We hypothesize that these CT markers (the BHS and BS) are surrogates for a phenomenon similar to that mediating the spot sign and that that this represents ongoing, multifocal, active bleeding within the hematoma.

In our study, the BHS and BS exhibited favorable abilities to predict the EHE, with the BS performing slightly better than the BHS. We also directly compared the diagnostic performances of the BHS and BS and found that the AUC for the BS was significantly higher than the AUC for the BHS in predicting HE. To explain these results, we compared hematoma growth between those with and without these CT markers, according to baseline hematoma volumes. Heterogeneous density on NCCT (39) and a larger baseline hematoma volume (26) may indicate active hemorrhage or multiple bleeding vessels, which lead to an increased risk of the BS. The BS and BHS were found to be associated with a higher risk of postoperative rebleeding in sICH patients undergoing surgical intervention (33, 40, 41), and this also indirectly confirmed the underlying mechanisms of HE. Therefore, the presence of the BS combined with larger baseline hematoma volume could identify those patients most likely to benefit from anti-HE therapies.

HE was associated with poor functional outcome (4, 42), which can be influenced by therapy (43–45). However, it was unclear if the absolute growth led to poor outcomes in individuals. There is no universal consensus on a clinically meaningful definition of HE (11, 12, 46, 47). At present, >6 ml (48, 49) or >12.5 ml (9) absolute growth is the widely used definitions of HE. According to the method used by Youden, Dowlatshahi et al. (25) proposed the best cutoff value for absolute growth to be >3 ml in predicting poor outcome. Absolute hematoma growth was categorized into four groups on a continuous scale in the previous study, which, compared to the binary classification of HE, provided additional stratification by volume for the risk of poor outcome following HE.

In a previous study, Morotti et al. (50) established the BAT score (including the CT BS and time of baseline NCCT <2.5 h), which could predict HE and emphasized that the time from onset to NCCT <2.5 h was significantly correlated with HE. Ovesen et al. (51) indicated that the shorter the time from symptom onset to baseline CT, the higher the incidence of HE, especially in patients with a time to baseline CT of <3 h. A recent meta-analysis (2) found that the rate of decline was steepest 0.5–3 h after ICH symptom onset and that the median time from symptom onset to baseline CT scan was 2.2 (IQR 1.3–4.2). The proportion of patients with HE (defined as >6 ml) was 22%. Our study showed that time to baseline CT scan (median 3 h, IQR 2–4) was an independent predictor of EHE and that the incidence of HE (also defined as >6 ml) was 15.9% in patients with ICH. However, some patients were at or beyond the time of greatest risk of HE in our cohort. A small number of patients who required early surgical intervention or abandonment of treatment were excluded. Moreover, patients with missing data were likely to have had more severe deficits or died early and might be considered to have a higher incidence of HE; therefore, excluding them contributed to underestimating the real performance. We recognized the potential for selection bias to influence our results. Therefore, we adjusted for these and other known confounding variables in the multivariate model.

Many patients with sICH are likely to have a poor outcome irrespective of the presence or absence of HE, and this is possibly due to the hematoma location, intraventricular extension, age, or large initial hematoma volume; none of these factors can be modified. There has been hope that poor outcomes and mortality related to high-volume hemorrhage could be ameliorated by interventions that aim to lower hemorrhage volume in patients with sICH. The results from the INTERACT1 study reaffirmed the importance of HE as a determinant of poor outcome and mortality. In our study, we found that an increase in the EHE was associated with an increase in the risk of a poor outcome. Conversely, smaller hematomas were less likely to produce poor outcomes, which is consistent with prior studies (8). Our study reinforces the association between EHE and clinical outcomes in patients with sICH. The most plausible explanation for these findings is that the absolute HE is directly proportional to the volume of the brain tissue destroyed. In addition, with the popularity of minimally invasive neurosurgical techniques, patients with a high risk of HE could receive more intensive neurological monitoring, and targeted surgical hematoma evacuation. Stratification of HE risk has important implications for the clinical care of patients with sICH.

Our study has several limitations. First, our study results were derived from a retrospective single-center analysis and lack data from formal orders for withdrawal of care, which require further validation. Second, the proportion of Chinese participants with deep ICH was higher than that among Western populations, which may limit the generalizability of our findings to other ethnic groups. Third, emergency CTA was not performed in all patients from our study population, which may have affected our results. Moreover, the presence of an underlying macrovascular abnormality was not detected at the time of the baseline NCCT. Thus, using the NCCT features to predict poor outcome and HE possibly influenced early treatment decisions. Fourth, given the retrospective design and in a study investigating emergencies, standardized therapeutic procedures were difficult to establish, and we were also not able to fully account for acute-phase therapies that may affect hematoma growth. Fifth, the exact timing of the follow-up CT was at the discretion of the clinical physician based on clinical judgment, patient preference, and treatment need, but we attempted to minimize this effect as much as possible by adjusting for the time of follow-up CT in the HE models. Finally, functional outcomes were assessed at discharge, which may not reflect the long-term outcomes for patients with sICH. Future studies using larger samples are needed to support our findings.

## Conclusions

The CT BS showed a better performance in predicting the EHE in patients with sICH than did other NCCT markers. These markers may help identify patients at a high risk of HE and provide a potential target for anti-HE treatments for patients with acute sICH. Meanwhile, the lack of these markers may enable clinicians to identify patients at lower risk of HE being more suitable for admission to general neurology wards, especially in limited resource settings. Because patients without these markers appear to be relatively stable, anti-expansion treatment such as intensive blood pressure lowering or surgical intervention may offer little value.

## Data Availability Statement

The datasets generated for this study will be made available on request to the corresponding author.

## Ethics Statement

This study was performed in accordance with the recommendations of the Medical Ethics Committee of The First Affiliated Hospital of Wenzhou Medical University. Written informed consent from all participation was waived. The protocol was approved by the Medical Ethics Committee of The First Affiliated Hospital of Wenzhou Medical University.

## Author Contributions

YY and MZ contributed to the study conception and design. YY acquired the funding. MZ, JC, CZ, JL, CC, TZ, TX, and DZ collected the patients' data. CZ and MZ checked the data and performed statistical analyses. MZ drafted the article. YY and QC critically revised it. All authors reviewed the final manuscript and approved it to be submitted. We would like to thank Editage (www.editage.cn) for English language editing.

## Conflict of Interest

The authors declare that the research was conducted in the absence of any commercial or financial relationships that could be construed as a potential conflict of interest.
